# Developmental trajectories from day 2 to day 3 refine ranking and selection of cleavage-stage embryos

**DOI:** 10.1007/s10815-026-03973-4

**Published:** 2026-07-25

**Authors:** Wen-jie Huo, Fei Peng, Chen Luo, Zhi-heng Chen, Qiu-xia Yan, Ling Sun, Cai-rong Chen, Song Quan, Xiao-cong Wang

**Affiliations:** 1 Department of Obstetrics and Gynecology, Nanfang Hospital, Southern Medical University, Guangzhou, China; 2 Department of Psychology, School of Public Health, Southern Medical University, Guangzhou, China; 3 Department of Psychiatry, Zhujiang Hospital, Southern Medical University, Guangzhou, China; 4 Center of Reproductive Medicine, Guangzhou Women and Children’s Medical Center, Guangzhou Medical University, Guangzhou, China; 5 Center for Reproductive Medicine, The Affiliated Qingyuan Hospital (Qingyuan People’s Hospital), Guangzhou Medical University, Qingyuan, China

**Keywords:** Developmental trajectory, Cell number, Embryo ranking, Blastulation, Clinical pregnancy rate

## Abstract

**Purpose:**

To determine whether day 2 to day 3 developmental trajectories improve day 3 embryo ranking and selection compared with day 3 cell number alone, thereby offering better prognostic information for cleavage-stage embryos, including fast-cleaving embryos whose developmental potential remains uncertain.

**Design:**

This retrospective multicenter study (2016–2024) developed a ranking framework using 27,002 embryos from one center and validated it in 1691 fresh day 3 single embryo transfers across three centers (maternal age ≤ 38 years; embryos derived from two pronuclei, with fragmentation ≤ 10%, symmetric, and no multinucleation for confounder control). Developmental trajectories combined day 2 and day 3 cell numbers. Mixed-effects logistic models assessed trajectory–blastulation associations; post hoc comparisons consolidated trajectories into four ranking categories. Validation used trend tests between ranking levels and pregnancy rates, multivariable mixed-effects logistic model to estimate adjusted odds ratios (aORs) between ranking levels, and AUC for pregnancy prediction.

**Results:**

Trajectory analysis reversed the advantage of day 3 8-cell over 9–16-cell embryos (30.7% vs. 24.2% high-quality blastulation; *P* < 0.001), with Normal_Fast (4 → 9–16) achieving the highest rate (40.7%), significantly higher than Normal_Normal (4 → 8; 35.1%; *P* < 0.001). Trajectories were consolidated into a four-category ranking: (1) Normal_Fast (4 → 9–16); (2) Normal_Normal (4 → 8); (3) Normal_Slow (4 → 6–7), Slow_Normal (< 4 → 8), Fast_Normal and Fast_Fast (> 4 → 8–16); and (4) all others. Ranking 1 had a higher likelihood of clinical pregnancy than ranking 2 (aOR = 2.06 [1.05–6.82]), although this validation was limited by sample size (*n* = 24). Trajectory outperformed day 3 cell number in predicting high-quality blastocyst (AUC 0.776 vs. 0.729; *P* < 0.001) and clinical pregnancy (AUC original 0.619 vs. 0.596, *P* = 0.02; down-sampled 0.682 vs. 0.618, *P* = 0.016).

**Conclusions:**

For women ≤ 38 years undergoing fresh day 3 single embryo transfer, developmental trajectories from day 2 to day 3 improve embryo ranking and selection with other morphological features controlled (two pronuclei, fragmentation ≤ 10%, symmetry, no multinucleation). The 4 → 9–16 pathway emerges as potentially optimal, though requiring further validation. This dual-day framework treats both cell numbers as interdependent markers, where day 2 status redefines the priority of day 3 cell number, rather than serving only as a secondary criterion.

**Supplementary Information:**

The online version contains supplementary material available at https://doi.org/10.1007/s10815-026-03973-4.

## Background

In vitro fertilization (IVF) has transformed reproductive medicine, yet selecting the most viable embryos remains a pivotal challenge [1, 2]. Embryo ranking is a critical step in embryo selection, which involves ordering usable embryos according to their developmental potential. Consequently, refining embryo ranking systems holds clinical importance, as it could increase pregnancy rates per transfer, shorten treatment duration, and reduce both the financial and emotional burden of repeated implantation failure [3, 4].

Two main embryo ranking systems exist based on final-day morphology prior to transfer: one for cleavage-stage embryos (day 3) and another for blastocysts (day 5/6). Despite the increasing adoption of blastocyst transfer, day 3 cleavage-stage transfer remains widely practiced in clinically controversial populations and in certain countries [5–8]. Yet day 3 embryos exhibit limited self-selection ability, and their morphology correlates weakly with pregnancy outcomes, making optimal embryo selection a persistent challenge in IVF [9]. Hence, refining the day 3 embryo ranking system is an important step forward.

International consensus has regarded day 3 cell number as the most important feature due to its reflection of the embryo’s developmental stage [2]. The current hierarchy is straightforward: 8-cell embryos are considered optimal, > 8-cell intermediate, and < 8-cell poor. However, this ranking scheme sometimes underestimates the potential of certain embryos. For instance, several studies suggest that > 8-cell embryos can achieve comparable or even superior clinical results to classic 8-cell embryos [10–13]. Such discrepancies indicate that day 3 cell number alone may not capture critical determinants of an embryo’s developmental competence.

Embryonic development is inherently a dynamic process. Embryos may reach similar morphology by day 3 through distinct developmental trajectories, and these trajectories may hold prognostic value. Time-lapse monitoring studies have demonstrated that developmental potential correlates more strongly with relative progression rates between key cleavage events than with absolute timelines [14–16]. Similarly, morphology analyses show that day 3 8-cell embryos differ in pregnancy outcomes depending on their preceding day 2 cell number [17]. Together, these findings underscore the value of integrating day 2 and day 3 developmental information to achieve a more comprehensive and accurate assessment of embryo competence.

This study aimed to develop and validate a novel embryo ranking system that integrates both day 2 and day 3 cell numbers in women ≤ 38 years, with other morphology features controlled (two pronuclei, fragmentation ≤ 10%, symmetry, no multinucleation). Using retrospective data from extended culture, we examined the relationship between developmental trajectories and embryo developmental potential and derived a ranking scheme accordingly. We then validated this system in a cohort of day 3 fresh single embryo transfer (SET) cycles. By incorporating developmental information across two consecutive days, our approach aims to provide a more comprehensive framework for day 3 embryo selection in matching clinical practice.

## Materials and methods

### Ethical approval

Ethical approval was granted by the ethics committees of all sites, including the Institutional Review Board of Nanfang Hospital (No. NFEC-2025–452), the Ethics Committee of Guangzhou Women and Children’s Medical Center (No. 2026-060A01), and the Ethics Committee of Qingyuan People’s Hospital (No. IRB-2026–010). The requirement for informed consent was waived because all data used here were retrospective and fully anonymized.

### Participants

**Fig. 1:**
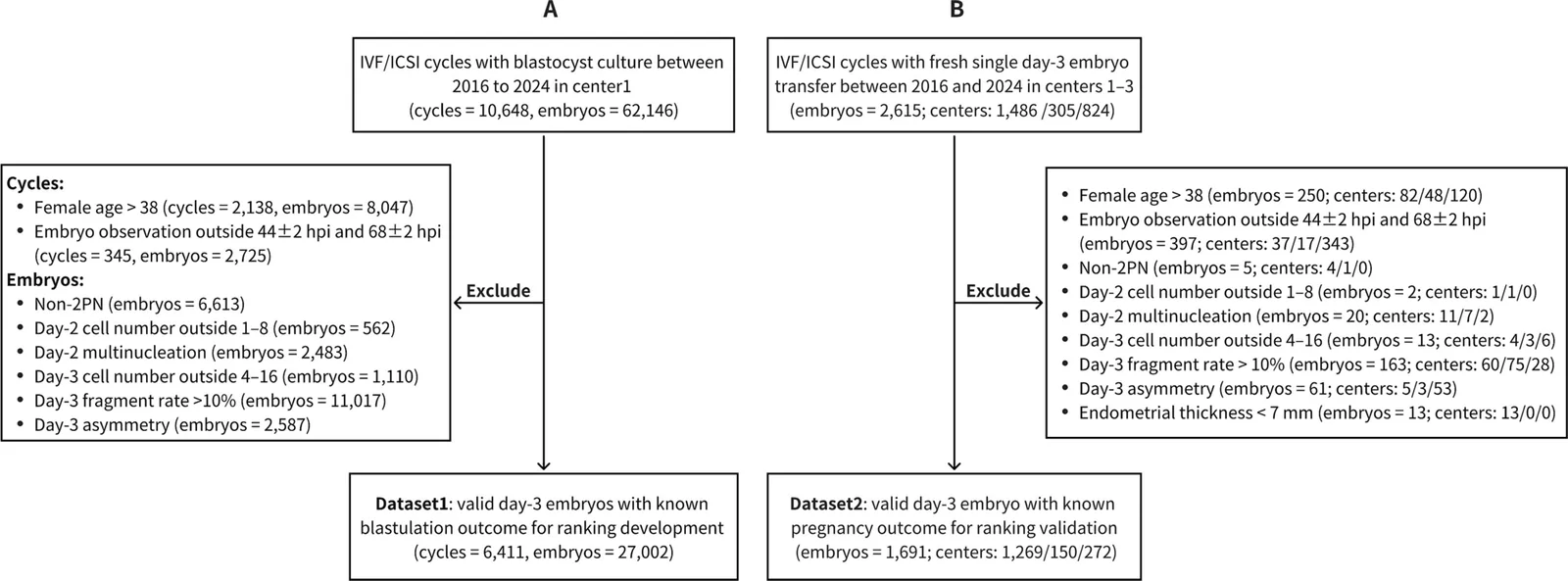
Flowchart of data inclusion and exclusion. **A** Dataset 1: extended-culture cycles for ranking development. **B** Dataset 2: fresh single day 3 embryo transfer cycles for ranking validation

This retrospective, multicenter cohort study was conducted at three tertiary-level hospitals in China. Dataset 1 (for ranking development) included autologous IVF/intracytoplasmic sperm injection (ICSI) cycles undergoing extended culture to day 5–6 at Nanfang Hospital, between January 2016 and April 2024. Exclusion criteria comprised (1) maternal age > 38 years; (2) observation outside defined time windows (day 2: 44 ± 2 h post-insemination [hpi]; day 3: 68 ± 2 hpi); (3) embryos with day 2 cell counts outside 1–8 or multinucleation; (4) day 3 cell counts outside 4–16, fragmentation > 10% or asymmetry; and (5) embryos from non-2 pronuclei (PN). A total of 62,146 embryos from 10,648 extended-culture cycles were initially included. After applying exclusion criteria, 27,002 embryos from 6411 extended-culture cycles remained for final analysis (Fig. 1A).

Dataset 2 (for ranking validation) included autologous IVF/ICSI-ET cycles involving day 3 SET with documented pregnancy outcomes, conducted between January 2016 and April 2024 at three sites: Nanfang Hospital, Qingyuan People’s Hospital, and Guangzhou Women and Children’s Medical Center. The same exclusion criteria as used for dataset 1 were applied, with the additional exclusion of cycles with endometrial thickness < 7 mm on transfer day. A total of 2615 SET cycles (center 1: 1486; center 2: 305; center 3: 824) were initially identified. After applying all exclusion criteria, 1691 cycles (center 1: 1269; center 2: 150; center 3: 272) remained for analysis (Fig. 1B).

### Treatment protocols

Ovarian stimulation followed standard GnRH-agonist or GnRH-antagonist protocols. A subset of patients received alternative stimulation approaches, including minimal stimulation, progestin-primed ovarian stimulation, and luteal phase ovarian stimulation. Final oocyte maturation was triggered when at least one follicle reached 18 mm, using 2000–10000 IU hCG (Livzen, China) or 250 μg Ovidrel (Merck-Serono, Switzerland), with or without the addition of 0.2 mg triptorelin (Decapeptyl, Ferring, Switzerland). Oocyte retrieval occurred 34–36 h after triggering under transvaginal ultrasound.

Fertilization was achieved by conventional IVF or ICSI. After insemination, embryos were cultured individually in pre-equilibrated cleavage medium (Vitrolife G-1) under mineral oil (Vitrolife OVOIL) at 37 °C, 6% CO_2_, and 5% O_2_, with a culture volume of 20–30 µL per drop in incubators (Labotect). At 44 ± 2 hpi (day 2) and 68 ± 2 hpi (day 3), blastomere count, fragmentation, symmetry, and multinucleation were assessed under an inverted microscope at 200–400 × magnification and recorded by senior embryologists with regular quality control audits to ensure interoperator reliability.

Fresh day 3 embryo transfer was the routine strategy when all-blastocyst culture risked total arrest (e.g., limited embryo number), provided there was no high risk of ovarian hyperstimulation syndrome or other contraindications. Accordingly, the development cohort included both all-embryo extended-culture cycles (in which all eligible embryos were extended-cultured) and partial extended-culture cycles (in which, after retaining 1–2 usable day 3 embryos for transfer per routine clinical criteria, selected surplus embryos were cultured further to day 5 or day 6), in blastocyst medium (Vitrolife G-2). Embryos that reached the blastocyst stage were then graded using Gardner criteria (expansion graded 1–6; inner cell mass and trophectoderm graded A–C). High-quality blastocyst was defined as ≥ 4 BB on day 5. Usable blastocyst was defined as ≥ 3 CC on day 5 or day 6. Laboratory protocols were consistent across centers and over time (2016–2024), except for assisted hatching on fresh day 3 embryo transfers: not performed in centers 1 or 3, but used in center 2 for embryos with abnormal zona pellucida.

### Feature collection

Day 2 cell number was categorized into three categories: slow (1–3-cell), normal (4-cell), and fast (5–8-cell). Day 3 cell number was classified into four categories: very slow (4–5-cell), slow (6–7-cell), normal (8-cell), and fast (9–16-cell). The two features were combined to construct the trajectory variable, resulting in 12 factors (3 × 4). For example, an embryo with 1–3 cells on day 2 and 4–5 cells on day 3 was classified as “slow_very slow” and so forth.

Other recorded embryo morphology features included pronuclear status on day 1 (2PN vs. non-2PN), multinucleation on day 2, fragmentation percentage on days 2 and 3 (0–5%, 10%, > 10%), and symmetry on days 2 and 3 (symmetric vs. asymmetric). Patient demographics comprised female age, male age, body mass index, infertility type (primary vs. secondary), and etiology (female, male, combined, or unexplained). Cycle characteristics involved the stimulation protocol (agonist, antagonist, or nonconventional), fertilization method (IVF, ICSI), and the documented times of insemination, day 2 observation, and day 3 observation, respectively. For dataset 2, endometrial thickness on transfer day was also collected.

### Outcome measures

The usable blastulation rate was defined as the number of usable blastocysts (≥ 3 CC on day 5 or day 6) divided by total number of embryos undergoing extended culture. The high-quality blastulation rate was defined as the number of high-quality blastocysts (≥ 4 BB on day 5) divided by total number of embryos undergoing extended culture. The clinical pregnancy rate (CPR) was defined as the number of transfer cycles with at least one gestational sac and fetal heartbeat detected at 4 weeks, divided by the total number of embryo transfer cycles.

### Statistical analysis

Continuous variables are presented as mean ± standard deviation (SD), while categorical variables are presented as percentages. Differences were assessed using *t* tests for continuous variables and *χ*^2^ tests for categorical variables. Statistical significance was defined as a two-tailed *P* value < 0.05.

The independent association between developmental trajectories and blastulation was assessed using a multivariable mixed-effects logistic regression model, adjusting for female age, treatment strategy, fertilization method, day 2 symmetry, and day 3 fragmentation, with patient identifier and observation time as random effects. This set of confounders was selected by comparing all possible variable combinations via a best-subset approach and retaining the model with the minimum Akaike information criterion (AIC). The effect size of the developmental trajectory on blastulation is presented as adjusted odds ratios (aORs) with 95% confidence intervals (CIs). A hierarchical order was established via Tukey post hoc comparisons, with trajectories showing no significant difference consolidated into the same rank, resulting in a four-tier system.

For validation, the association between the predefined ranking (1 to 4) and pregnancy rate within each center was first assessed using the Cochran-Armitage test for trend. To estimate the adjusted effect of each ranking category across all centers, a mixed-effects multivariable logistic regression model was fitted, with ranking as a fixed categorical effect, center as a random intercept, and adjustment for female age, endometrial thickness and day 3 fragmentation (selected using the same minimum AIC approach described above). Results are reported as aOR with 95% CI.

The discriminatory power of the trajectory-based versus the conventional day 3 cell number ranking for predicting blastulation and clinical pregnancy was evaluated by the area under the receiver operating characteristic curve (AUC). Due to differences in sample size, model performance was internally validated using an 8:2 train-test split for blastulation prediction (larger cohort) and a 6:4 split for clinical pregnancy prediction (smaller cohort). The statistical significance of differences in test-AUC between paired models was assessed using DeLong’s test, which is specifically designed for comparing receiver operating characteristic (ROC) curves.

All statistical analyses were performed using R software (version 4.4.1, R Foundation for Statistical Computing). The following packages were utilized for specific analyses: the stats package for descriptive statistics, chi-square tests, and logistic regression; the MASS package for stepwise variable selection via the stepAIC function; the lme4 package for fitting mixed-effects models; the emmeans package for post hoc pairwise comparisons with Tukey adjustment; and the pROC package for ROC curve analysis and DeLong’s test.

## Results

### Development of trajectory-based embryo ranking system

**Fig. 2:**
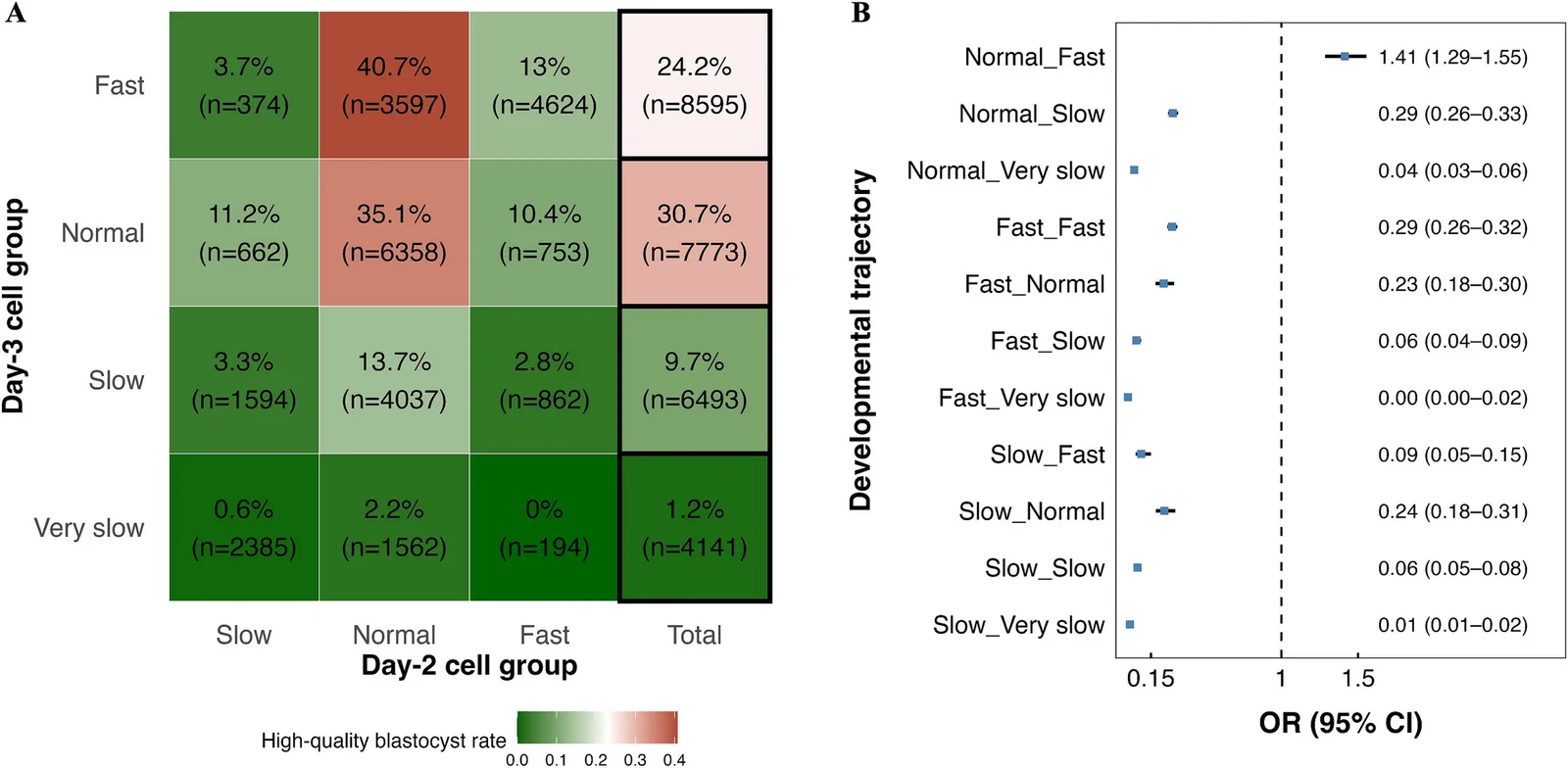
Impact of developmental trajectory on high-quality blastulation. **A** Heatmap showing high-quality blastulation rates for each developmental trajectory, defined by day 2 cell groups (horizontal axis: slow [1–3 cells], normal [4 cells], fast [5–8 cells], and total [1–8 cells]) and day 3 cell groups (vertical axis: very slow [4–5 cells], slow [6–7 cells], normal [8 cells], fast [9–16 cells]). Each tile displays the sample size (*n*) and high-quality blastulation rate (%), which is also indicated by color intensity. **B** Forest plot showing the adjusted impact of each trajectory on blastocyst formation. Trajectory labels (e.g., slow_fast) indicate the transition from day 2 to day 3 cell number groups. Blue squares represent odds ratios (ORs), with black error bars denoting 95% confidence intervals; the normal_normal trajectory serves as the reference (OR = 1)

Of the 27,002 day 3 embryos that underwent extended culture, 17,685 (65.5%) formed usable blastocysts and 5141 (19.0%) formed high-quality blastocysts. To visualize the relationship between developmental trajectories and blastulation outcome, we constructed a two-dimensional plot of day 2 and day 3 cell number, with sample size and high-quality blastulation rate shown for each combination (Fig. 2A). Detailed patient and cycle characteristics are summarized in Table 1. Interestingly, the apparent advantage of day 3 8-cell over 9–16-cell embryos (30.7% vs 24.2%; *P* < 0.001) was reversed when day 2 cell number was considered within developmental trajectories. Specifically, embryos following the normal_normal trajectory (4-cell → 8-cell) exhibited a high-quality blastulation rate of 35.1%, which was significantly lower than those with normal_fast trajectory (4-cell → 9–16-cell; 40.7%; *P* < 0.001) but significantly higher than those with slow_fast and fast_fast trajectories (non-4-cell → 9–16-cell; 3.7% and 13%; both *P* < 0.001).

**Table 1:** Baseline demographics and characteristics of embryos undergoing extended culture (dataset 1, *n* = 27,002)

Features	Mean ± SD/*n* (%)
Female age	31.4 ± 3.9
BMI	21.6 ± 2.9
Male age	33.8 ± 5.0
Infertility type (primary)	10,531 (39.0%)
Infertility cause	
Female	19,765 (73.2%)
Male	4050 (15.0%)
Combined	1485 (5.5%)
Unexplained	1702 (6.3%)
Stimulation protocol	
Agonist	14,432 (53.4%)
Antagonist	11,045 (40.9%)
Nonconventional	1525 (5.6%)
Fertilization method	
IVF	20,560 (76.1%)
ICSI	6442 (23.9%)
Day 2 cell number	
1–3	5015 (18.6%)
4	15,554 (57.6%)
5–8	6433 (23.8%)
Day 2 fragmentation rate (0–5%)	20,040 (74.2%)
Day 2 symmetry	25,973 (96.2%)
Day 3 cell number	
4–5	4141 (15.3%)
6–7	6493 (24.0%)
8	7773 (28.8%)
9–16	8595 (31.8%)
D3 fragmentation rate (< 10%)	18,139 (67.2%)
High-quality blastulation (D5 ≥ 4 BB)	5141 (19.0%)
Usable blastulation (D5/6 ≥ 3 CC)	17,685 (65.5%)
Blastulation (expansion grade 1–6)	21,819 (80.8%)

*SD* standard deviation, *BMI* body mass index, *IVF* in vitro fertilization, *ICSI* intracytoplasmic sperm injection

**Fig. 3:**
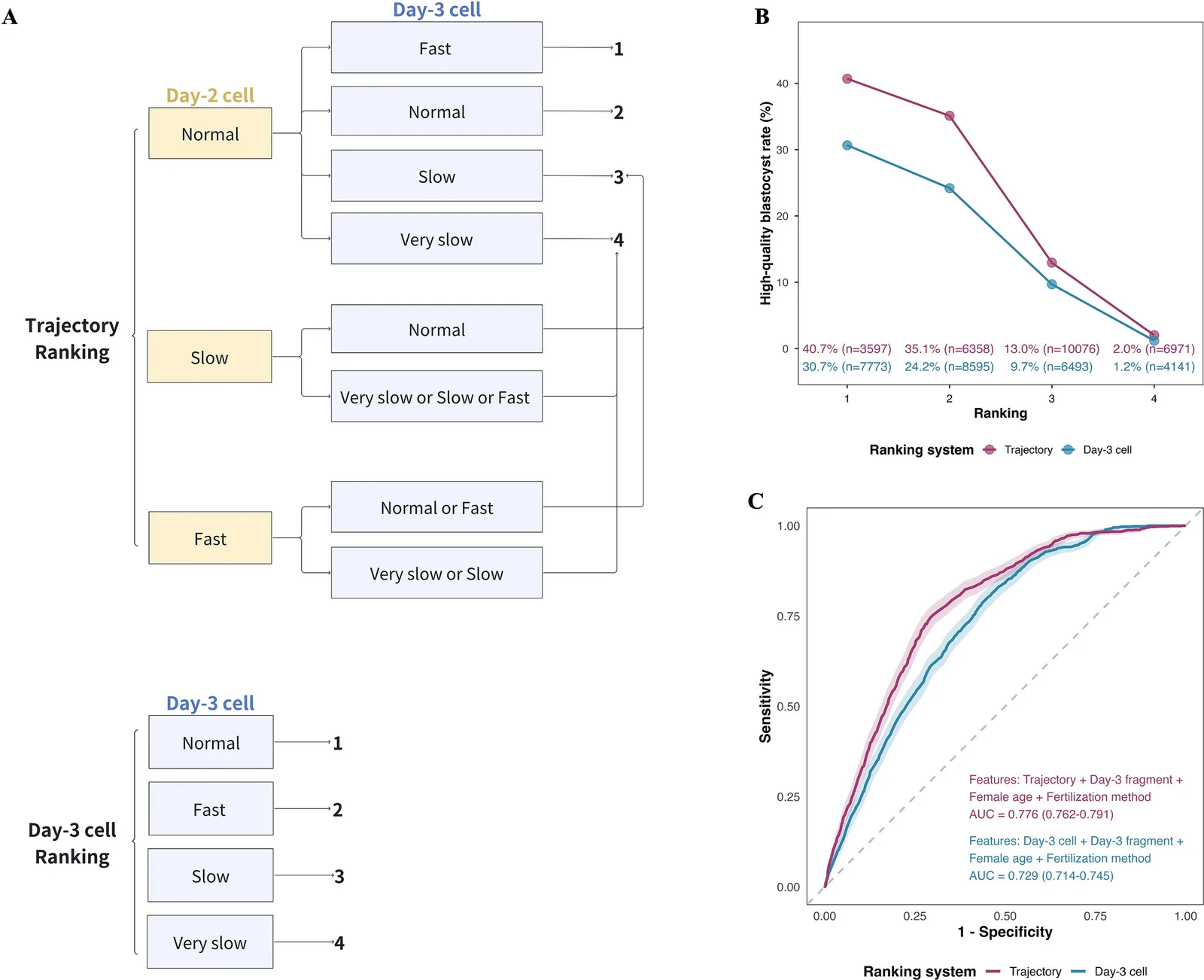
Comparison of the trajectory-based ranking system versus the conventional day 3 cell number system. **A** Schematic representation of the four-category trajectory-based ranking (top) and the conventional day 3 cell number ranking (bottom). **B** Comparison of high-quality blastulation rates between the two ranking systems. **C** Receiver operating characteristic (ROC) curves comparing the blastulation prediction performance of the trajectory-based model and the day 3 cell number model on the test set

To assess the independent association between developmental trajectory and blastulation, we constructed a mixed-effects multivariable logistic model. After adjusting for female age, treatment strategy, fertilization method, and day 3 fragmentation, with patient identifier and observation time as random effects, the association remained significant (Fig. 2B). Post hoc pairwise comparisons revealed the following four-category trajectory-based ranking system: (1) normal_fast (4-cell → 9–16-cell); (2) normal_normal (4-cell → 8-cell); (3) normal_slow (4-cell → 6–7-cell), slow_normal (< 4-cell → 8-cell), and fast_normal/fast (> 4-cell → 8–16-cell); and (4) the remainders (Figs. 2B and 3A). Additionally, subgroup analyses by fertilization method (IVF vs. ICSI) showed consistent trajectory–blastulation outcome relationships and identical ranking orders (Supplementary Fig. 1), supporting the robustness of our findings across fertilization methods.

We further compared the trajectory-based ranking system with the conventional day 3 cell number system (four categories: 8, 9–16, 6–7, and 4–5 cells, Fig. 3A). The trajectory-based system demonstrated superior performance in identifying embryos with higher developmental potential, as shown by higher high-quality blastulation rates in its top ranks (ranking 1: 40.7% vs. 30.7%; ranking 2: 35.1% vs. 24.2%, both *P* < 0.001; Fig. 3B). Consistent with this ranking, the broader blastocyst outcome distribution showed that normal_fast maintained favorable overall outcomes, with a higher proportion of D5 high-quality blastocysts than normal_normal, while lower-ranked trajectories showed reduced usable blastocyst formation and increased developmental arrest (Supplementary Fig. 2). Moreover, replacing day 3 cell number with developmental trajectory in the predictive model improved the AUC from 0.729 to 0.776 (DeLong’s test, *P* < 0.001) on the test set after an 8:2 train-test (Fig. 3C). Collectively, these results indicate that the trajectory-based ranking system offers a more precise and reliable assessment of day 3 in vitro developmental potential than the traditional evaluation based on day 3 cell number alone.

### Validation of trajectory-based embryo ranking system

To validate whether the ranking system established on blastulation potential is valid for implantation outcome, we applied it to a multicenter cohort of 1691 embryos undergoing fresh cleavage-stage (day 3) SET (dataset 2). The overall clinical pregnancy rates were 53.7% (681/1269) in center 1, 44.7% (67/150) in center 2, and 44.1% (120/272) in center 3.

**Fig. 4:**
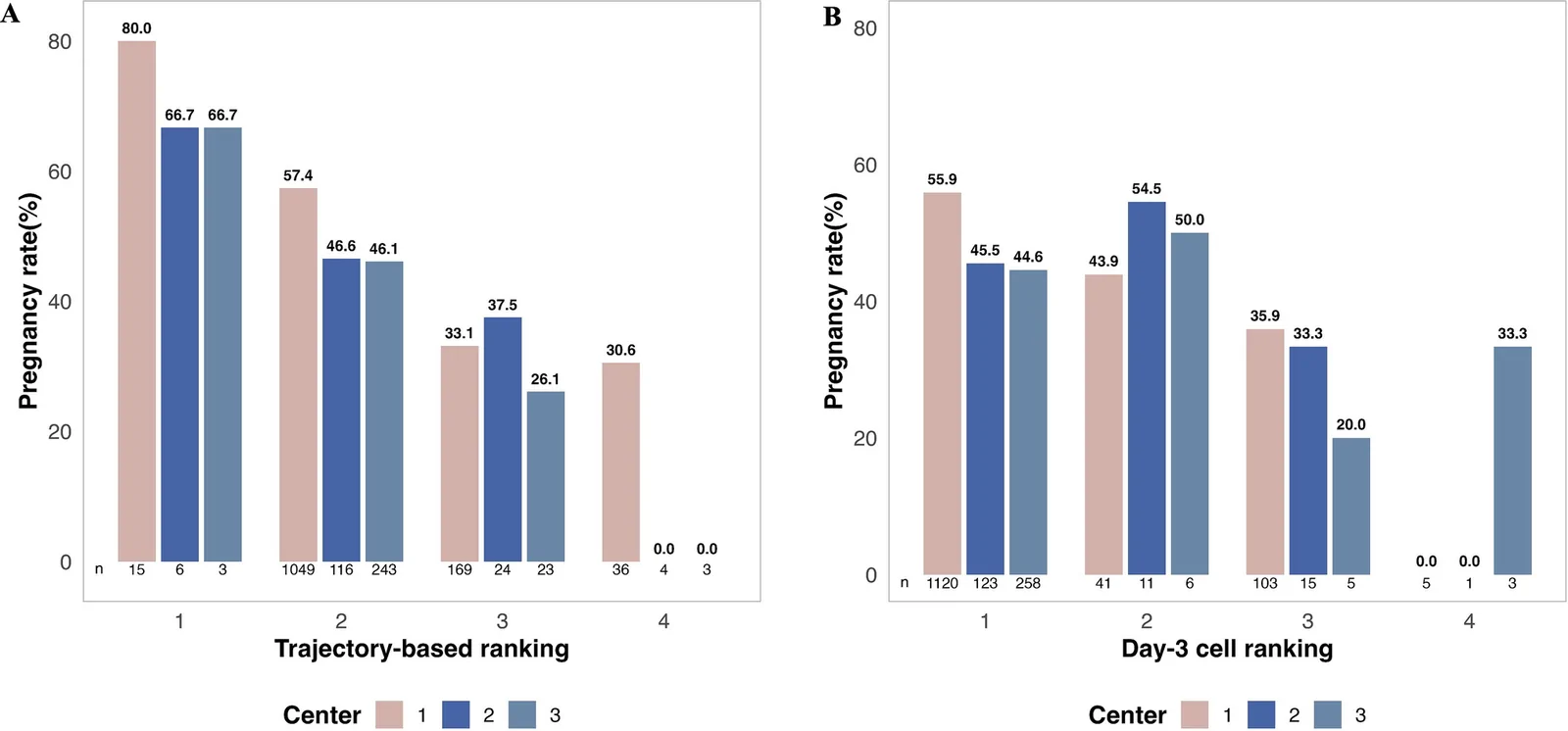
Multicenter clinical validation of the trajectory ranking system. **A** Association between trajectory ranking and pregnancy rates across centers. **B** Association between day 3 cell number ranking and pregnancy rates across centers

Embryos from each center were grouped according to the two ranking systems. A significant decreasing trend was observed between the trajectory-based ranking order (1 to 4) and clinical pregnancy rates across all centers (Fig. 4A, P for trend < 0.001, 0.038, and 0.01, respectively). In contrast, the conventional day 3 cell number ranking did not show a significant trend in two of the three centers (Fig. 4B, P for trend < 0.001, 0.37 and 0.39, respectively). Baseline characteristics are detailed in Table 2.

**Table 2:** Baseline demographics and characteristics of day 3 single embryo transfer cycles across three centers (dataset 2, *n* = 1691)

Features	Mean ± SD/*n* (%)		
	Center 1	Center 2	Center 3
Number of cycles	1269	150	272
Female age	31.5 ± 3.6	32.1 ± 3.7	31.4 ± 3.5
BMI	21.6 ± 2.9	21.7 ± 2.9	21.4 ± 2.9
Stimulation protocol			
Agonist	806 (63.5%)	98 (65.3%)	120 (44.1%)
Antagonist	448 (35.3%)	48 (32.0%)	135 (49.6%)
Nonconventional	15 (1.2%)	4 (2.7%)	17 (6.3%)
Endometrial thickness (mm)	10.9 ± 2.4	11.7 ± 2.7	10.8 ± 2.1
Day 2 cell number			
1–3	54 (4.3%)	6 (4.0%)	9 (3.3%)
4	1139 (89.8%)	135 (90.0%)	252 (92.7%)
5–8	76 (6.0%)	9 (6.0%)	11 (4.0%)
Day 2 fragmentation rate (0–5%)	1115 (87.9%)	83 (55.3%)	229 (84.2%)
Day 3 cell number			
4–5	5 (0.4%)	1 (0.7%)	3 (1.1%)
6–7	103 (8.1%)	15 (10.0%)	5 (1.8%)
8	1120 (88.3%)	123 (82%)	258 (94.9%)
9–16	41 (3.2%)	11 (7.3%)	6 (2.2%)
Day 3 fragmentation rate (< 10%)	1090 (85.9%)	69 (46.0%)	220 (80.9%)
Pregnancy rate	681 (53.7%)	67 (44.7%)	120 (44.1%)

*SD* standard deviation, *BMI* body mass index

To determine whether ranking 1 embryos were superior to ranking 2 embryos after accounting for confounders, we performed a pooled, multivariable analysis across centers, incorporating center as a random effect and adjusting for female age, endometrial thickness, and day 3 fragmentation (Table 3). Compared to ranking 2, ranking 1 was associated with a significantly higher likelihood of clinical pregnancy (aOR = 2.06, 95% CI 1.05–6.82), while rankings 3 and 4 were associated with significantly lower likelihoods (aOR = 0.43, 95% CI 0.31–0.58 and aOR = 0.30, 95% CI 0.15–0.59, respectively).

**Table 3:** Multivariable mixed-effects logistic regression analysis of pregnancy outcomes in day 3 embryo transfer cycles across multicenters (dataset 2)

Features	aOR	95% CI
Trajectory ranking		
1	2.06	1.05–6.82
2	Reference	Reference
3	0.427	0.31–0.58
4	0.296	0.15–0.59
Female age	0.98	0.95–1.00
Day 3 fragmentation		
0–5%	Reference	Reference
10%	0.776	0.60–1.01
Endometrial thickness	1.49	0.98–2.25

The model was fitted with center as a random effect, adjusting for trajectory ranking, female age, endometrial thickness, and day 3 fragmentation

*aOR* adjusted odds ratio, *CI* confidence interval

**Fig. 5:**
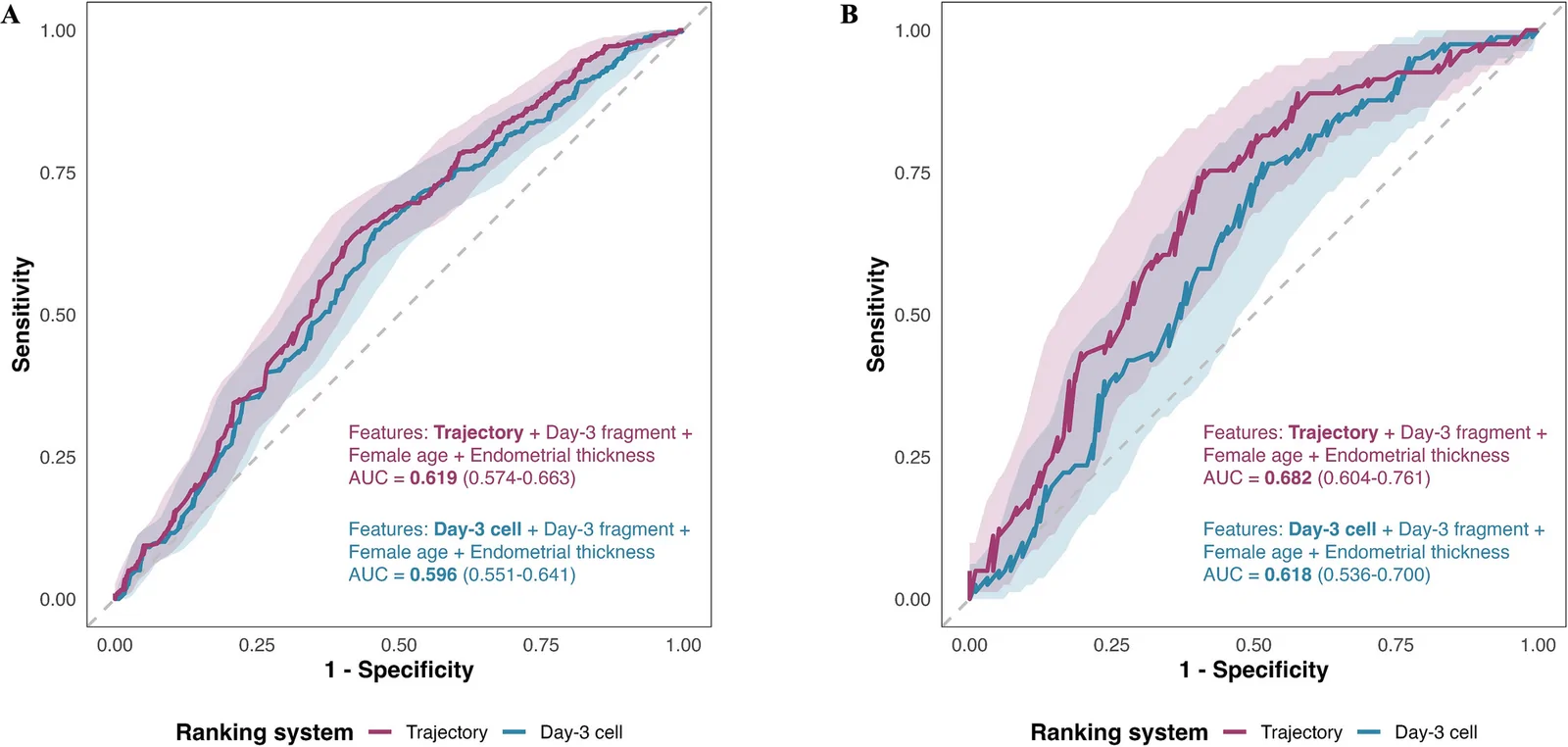
Receiver operating characteristic (ROC) curves comparing the pregnancy prediction performance of the trajectory-based model and the day 3 cell number model on the test set. **A** Original imbalanced test set. **B** Balanced subtest set after down-sampling ranking 2

Additionally, the overall predictive performance was superior for the trajectory‑based model. Its discriminative ability, measured by AUC on the test set (*n* = 676), was significantly higher than that of the day 3 cell number model (AUC 0.619 vs. 0.596; DeLong’s test, *P* = 0.02; Fig. 5A). However, the majority of embryos were concentrated in ranking 2, with sample sizes for rankings 1, 2, 3, and 4 being 14, 549, 96, and 17, respectively. To assess performance under balanced conditions, we down‑sampled ranking 2 (retaining 20%) to match the size of the other ranks and recalculated the AUCs. After down-sampling, both ranking systems’ AUC improved, with the trajectory-based system showing a greater advantage (AUC 0.682 vs. 0.618; DeLong’s test, *P* = 0.016; Fig. 5B).

## Discussion

This study introduces a trajectory-based framework for day 3 embryo ranking that incorporates both day 2 and day 3 cell numbers. Compared with conventional single-day morphology schemes, this approach provides a more accurate tool for embryo selection and offers a biologically grounded explanation for the variable performance of fast-cleaving embryos.

Literature suggests that rapid development rate has heterogeneous prognostic value: It may signify physiologically superior, coordinated cell cycle acceleration or, conversely, abnormalities such as aneuploidy and direct cleavage [18–20]. Although static morphology alone cannot directly distinguish these underlying causes, our restriction to embryos from females ≤ 38 years with two pronuclei, fragmentation ≤ 10%, symmetry, and no multinucleation serves as a morphological filter to enrich for biologically normal embryos [21, 22]. Thus, our ranking system is specifically applicable to embryos that pass this morphological filter. We posit that in cohorts with a higher prevalence of abnormalities, the performance of the ranking could change, as “fast” cleavage may more frequently signal an aberrant state. Therefore, the model should not be extrapolated to other contexts without further validation. Nevertheless, our ranking system aligns widely with the clinical goal of selecting the single best embryo from the available cohort of moderate- to high-quality embryos.

Day 3 embryo transfer has historically been associated with relatively modest pregnancy rates and limited prognostic value [9, 23, 24]. While part of this limitation reflects the biological constraints of early-stage embryos, it is also driven by the shortcomings of current grading and ranking systems [10, 11]. Our analysis demonstrates how developmental trajectories refine embryo ranking beyond what is possible with day 3 morphology alone: Among 9–16-cell embryos, the 4 → 9–16 pathway (rank 1) was associated with superior potential relative to the 4 → 8 (rank 2) benchmark, whereas non-4 → 9–16 (rank 3 or 4) embryos underperformed. Similarly, within the 8-cell group, the 4 → 8 trajectory outperformed the 4 → 6–7 (rank 3), with non-4 → 8 embryos (rank 3) showing comparable outcomes. This refined discrimination translated into superior overall predictive performance, as demonstrated by a higher AUC for both high-quality blastulation and pregnancy in the trajectory-based versus the conventional day 3 cell number ranking system.

The biological plausibility of this framework may lie in cell cycle dynamics. A typical cell cycle spans 10–12 h, and the 24-h interval between day 2 (44 hpi) and day 3 (68 hpi) allows for two consecutive mitotic divisions [25–28]. Deviations from this timing carry prognostic implications: Prolonged cycles often indicate checkpoint-mediated DNA repair, while overly compressed cycles risk incomplete genome synthesis [29, 30]. Our results align with such distinction between physiologic and pathologic kinetics. For example, embryos following the 4 → 9–16 trajectory completed two division cycles within the day 2 to day 3 interval and showed the best developmental potential. Although this pace is somewhat accelerated relative to the 4 → 8 (one division cycle), it appears to represent a physiologically rapid but orderly progression, consistent with efficient genome replication and intact cell cycle control [18]. However, this interpretation warrants time-lapse validation, as our static observations cannot distinguish normal from abnormal rapid cleavage (e.g., direct cleavage).

For day 2 non-4-cell embryos, developmental trajectory was again informative: Physiologic proliferation (1–2 cell cycles, e.g., < 4 → 8 and > 4 → 8–16, rank 3) yielded moderate outcomes, while outcomes deteriorated from overly slow (< 1 cell cycle, e.g., > 4 → 4–7, rank 4) or fast progression (> 2 cell cycles, e.g., < 4 → 9–16, rank 4). Together, these findings suggest that developmental trajectories capture the difference between physiologically rapid and pathologically compressed cycles, providing a mechanistic basis for interpreting the prognostic heterogeneity of fast-cleaving embryos.

The idea of jointly using day 2 and day 3 cell counts to define a top-quality embryo dates back to 1999 [31]. However, due to limited sample size and insufficient statistical power and validation, this pioneering idea did not gain much traction in clinical research. Later studies on “fast‐cleaving” embryos produced conflicting results [10, 11, 32], likely because day 2 status was not accounted for. Our findings help clarify these inconsistencies by showing that the prognostic value of rapid cleavage depends critically on whether it arises from a normal 4-cell baseline or from atypical states.

These insights carry both clinical and methodological implications. Clinically, the updated Istanbul consensus advances the timing of day 3 observation, potentially reclassifying some embryos previously considered fast-cleaving as normal [2]. However, this refinement does not fundamentally overcome the limitation of single time point assessment, still risking the selection of suboptimal 8-cell embryos and the omission of truly competent fast-cleaving embryos. The ASEBIR ranking system incorporates day 2 cell count but designates the 4 → 7–8 cell trajectory as optimal [1], a benchmark not supported by our findings. Collectively, our dual-day framework offers a more dynamic perspective that could reduce the misclassification of physiologically rapid cleavers and broaden the pool of optimal embryos suitable for fresh transfer. Regarding clinical feasibility, our framework is designed for laboratories performing routine day 2 assessment, offering an actionable refinement that integrates day 2 cell counts into day 3 embryo ranking without altering the workflow.

Methodologically, our findings highlight the importance of extended-culture datasets in developing robust ranking systems. Unlike transfer datasets, which are biased toward embryos already selected for transfer, blastulation datasets capture greater morphological diversity and are less affected by nonembryonic confounders. In our study, high-quality blastulation potential aligned well with pregnancy outcomes, reinforcing their utility as a training ground for pregnancy prediction models. As computer vision plays an expanding role in assisted reproduction [33], access to such large-scale datasets will be essential for building intelligent embryo selection algorithms.

Despite the study’s potential contributions, several limitations merit consideration. First, the developmental cohort (dataset 1) included both all-embryo and partial extended-culture cycles. In partial cycles, embryos entering extended culture were clinically selected, while embryos retained for day 3 transfer were not assessed for blastocyst outcomes. This may have introduced selection bias; therefore, the trajectory–blastocyst associations should be interpreted as extended-culture outcomes, rather than fully unbiased estimates for all day 3 embryos. Second, the validation cohort was overwhelmingly concentrated in the 4 → 8 group, with limited sample sizes in other trajectory groups, especially the top-ranked embryos (4 → 9–16), which may have reduced the reliability despite significant differences. The central paradox is that the 4 → 9–16 embryos were seldom transferred clinically, because they deviate from clinical embryo selection consensus. We therefore intend this work to serve as a preliminary exploration to draw attention to the 4 → 9–16 trajectory, while emphasizing that its potential superiority should be interpreted with caution and requires further confirmation. Third, precise timing of cleavage events and identification of abnormal cleavage patterns (e.g., direct cleavage) require time-lapse systems, which were not available in our settings. Future validation in time-lapse laboratories is warranted. Fourth, we excluded patients > 38 years and restricted to embryos with otherwise favorable morphology (two pronuclei, fragmentation ≤ 10%, symmetric, no multinucleation), without incorporating other transfer strategies or clinical outcomes. Therefore, our ranking system applies only to fresh day 3 single embryo transfer cycles in young patients with such filtered embryos and should not be extrapolated to other settings without further validation.

## Conclusions

In conclusion, developmental trajectories offer a more accurate and mechanistically informed approach to day 3 embryo ranking than reliance on day 3 cell number alone. The 4 → 9–16 trajectory emerged as a potentially optimal pathway, demonstrating that incorporating day 2 developmental context clarifies longstanding controversies over day 3 fast-cleaving embryos. By aligning embryo ranking with developmental process, this trajectory-based framework may refine embryo selection, offer a basis for improving pregnancy rates in fresh day 3 transfer cycles, and also inform the design of artificial intelligence tools.

## Supplementary Information

Below is the link to the electronic supplementary material.

ESM 1 (PDF 720 KB)

ESM 2 (PDF 802 KB)

## Data Availability

The datasets analyzed during the current study are not publicly available due to ethical restrictions that prohibit direct distribution of patient data but are available from the corresponding author on reasonable request.
