# Supplementary material for: Developmental trajectories from day 2 to day 3 refine ranking and selection of cleavage-stage embryos
Source: J Assist Reprod Genet. 2026 Jul 25;43(8):2581–92. doi: 10.1007/s10815-026-03973-4 (PMC13598021; doi:10.1007/s10815-026-03973-4)
Supplement: Supplementary file 1 [file 10815_2026_3973_MOESM1_ESM.pdf]

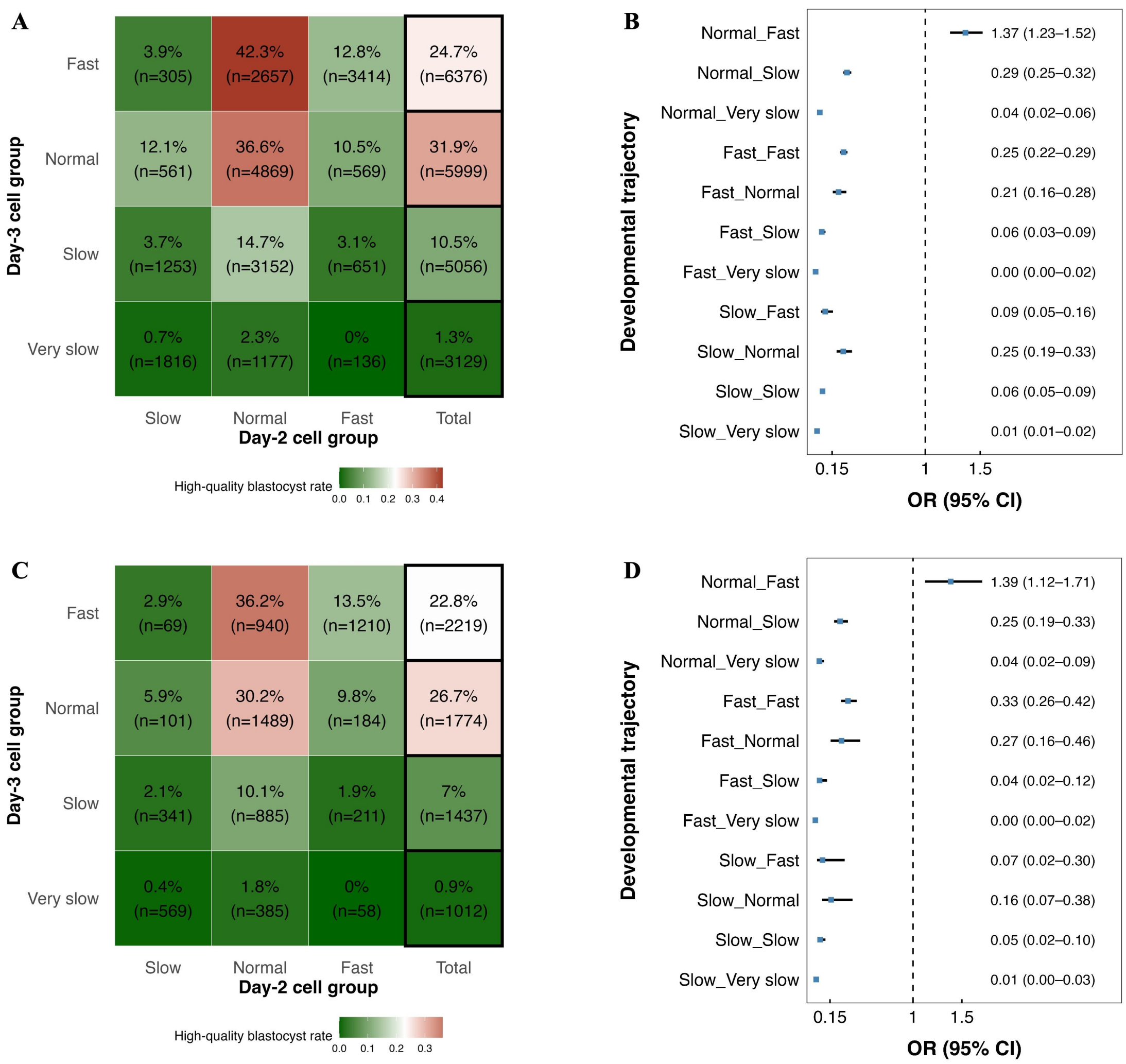

Supplementary Figure 1. Impact of developmental trajectory on high-quality blastulation, stratified by fertilization method (IVF vs. ICSI). (A, C) Heatmaps showing high-quality blastulation rates by Day-2 and Day-3 cell groups. Each tile shows sample size (n) and rate (%), with color intensity indicating rate. (A) IVF; (C) ICSI. (B, D) Forest plots of adjusted odds ratios (ORs) for each trajectory (Normal_Normal as reference). Blue squares = odds ratios (ORs); black error bars = 95% confidence intervals. (B) IVF; (D) ICSI.
