# Supplementary material for: Developmental trajectories from day 2 to day 3 refine ranking and selection of cleavage-stage embryos
Source: J Assist Reprod Genet. 2026 Jul 25;43(8):2581–92. doi: 10.1007/s10815-026-03973-4 (PMC13598021; doi:10.1007/s10815-026-03973-4)
Supplement: Supplementary file 2 [file 10815_2026_3973_MOESM2_ESM.pdf]

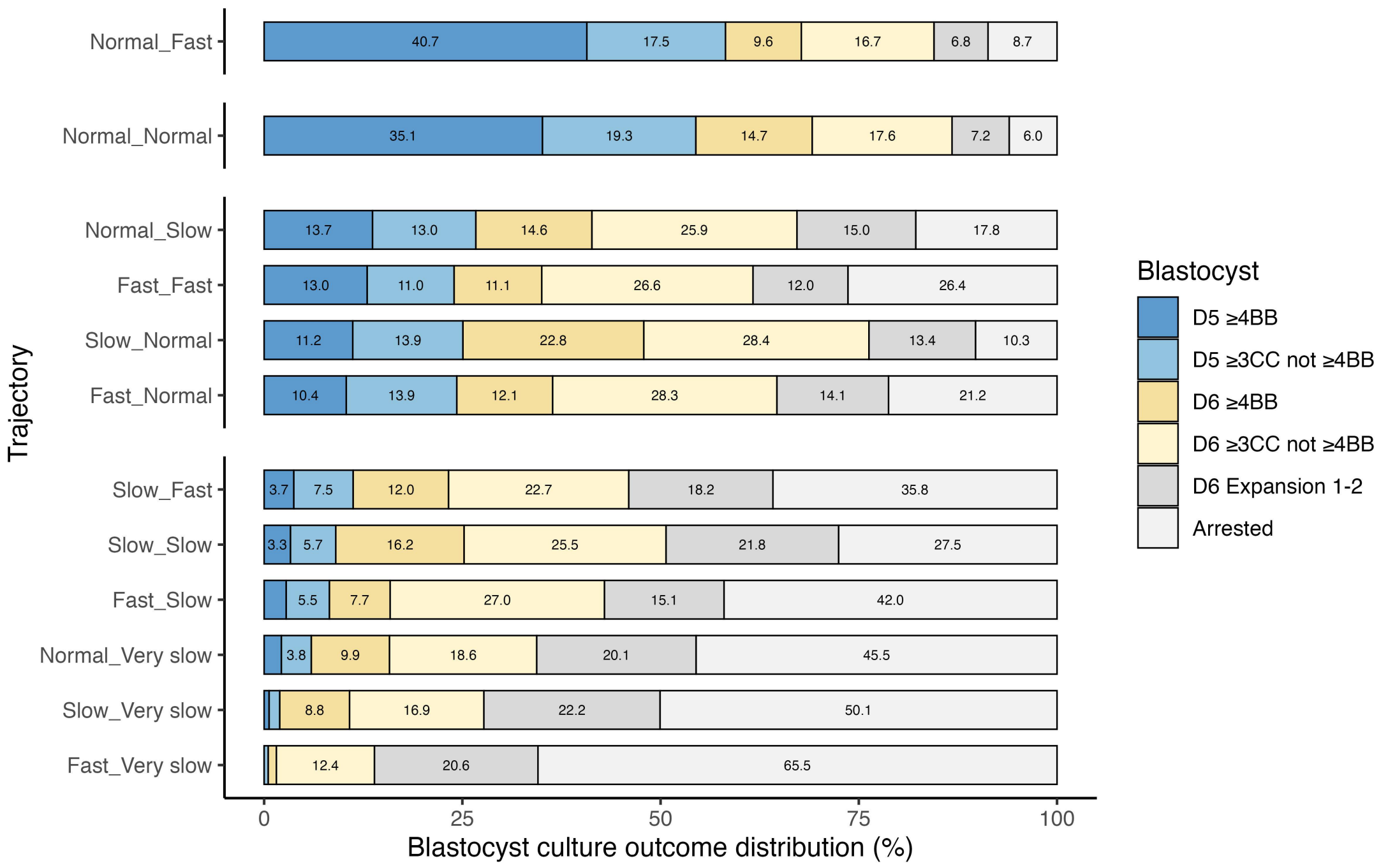

Supplementary Figure 2. Blastocyst outcome distribution by developmental trajectory. Trajectories are ordered top to bottom by decreasing high-quality blastocyst rate and grouped by the four-tier ranking. Each trajectory is a stacked bar chart. Grades (ordered from best to worst): D5 ⩾4BB, D5 ⩾3CC (not ⩾4BB), D6 ⩾4BB, D6 ⩾3CC (not ⩾4BB), D6 expansion 1–2, arrested. Usable blastocyst rate = sum of first four grades; total blastulation rate = sum of first five grades. Percentages <3% are not labeled.
